# Differentiation of preadipocytes and mature adipocytes requires PSMB8

**DOI:** 10.1038/srep26791

**Published:** 2016-05-26

**Authors:** Hideki Arimochi, Yuki Sasaki, Akiko Kitamura, Koji Yasutomo

**Affiliations:** 1 Department of Immunology and Parasitology, Graduate School of Medicine, Tokushima University, Japan; 2 JST, AMED-CREST, Japan

## Abstract

The differentiation of adipocytes is tightly regulated by a variety of intrinsic molecules and also by extrinsic molecules produced by adjacent cells. Dysfunction of adipocyte differentiation causes lipodystrophy, which impairs glucose and lipid homeostasis. Although dysfunction of immunoproteasomes causes partial lipodystrophy, the detailed molecular mechanisms remain to be determined. Here, we demonstrate that Psmb8, a catalytic subunit for immunoproteasomes, directly regulates the differentiation of preadipocytes and additionally the differentiation of preadipocytes to mature adipocytes. Psmb8^−/−^ mice exhibited slower weight gain than wild-type mice, and this was accompanied by reduced adipose tissue volume and smaller size of mature adipocytes compared with controls. Blockade of Psmb8 activity in 3T3-L1 cells disturbed the differentiation to mature adipocytes. Psmb8^−/−^ mice had fewer preadipocyte precursors, fewer preadipocytes and a reduced ability to differentiate preadipocytes toward mature adipocytes. Our data demonstrate that Psmb8-mediated immunoproteasome activity is a direct regulator of the differentiation of preadipocytes and their ultimate maturation.

Adipocyte differentiation requires tightly regulated transcription factors, including members of the CCAAT/enhancer-binding protein family, sterol regulatory element binding protein-1 and the nuclear receptor peroxisome proliferator-activated receptor γ^1^,^2^,^3^. These transcription factors help to express the genes that promote the differentiation of preadipocytes to mature adipocytes. Other adipogenic proteins, including fatty acid synthase, lipid droplet associated protein perilipin and adipophilin, are also required for the maturation of adipocytes^4^,^5^.

We and other groups have found that mutations in the *PSMB8* gene lead to JASL^6^, Nakajo-Nishimura syndrome^7^, CANDLE syndrome^8^ and JMP syndrome^9^. These diseases are characterized by high fever, nodular erythema, splenomegaly and lipodystrophy. The PSMB8 protein is a catalytic subunit in immunoproteasomes^10^, and a missense mutation causes an assembly defect in JASL patients^6^,^7^. This defect reduces proteasome activity, resulting in the accumulation of ubiquitin-coupled proteins^6^. The progressive lipodystrophy in JASL is dominant in upper or lower extremities, as the fatty tissue in the trunk is less affected. We have reported that downregulation of the *Psmb8* gene disturbed the differentiation of preadipocytes to mature adipocytes using 3T3-L1 cell lines *in vitro;* similar observations for *PSMB8* were made with human preadipocytes^6^. Furthermore, we have shown that inhibition of murine Psmb8 functionin adipose tissue reduced the adipose tissue volume *in vivo*^6^. However, it remains to be determined how immunoproteasomes control the differentiation of preadipocytes or the size of adipocytes *in vivo*.

Here, we investigated the differentiation of adipocytes in Psmb8-deficient (Psmb8^−/−^) mice. Psmb8^−/−^ mice had slower weight gain than control mice, with a reduced volume of adipose tissue and reduced adipocyte size. Psmb8^−/−^ mice had fewer preadipocyte precursors and preadipocytes, and Psmb8 deficient preadipocytes had a lower capacity to differentiate into mature adipocytes. Our data indicate that Psmb8-mediated immunoproteasome activity is a crucial regulator not only for the differentiation of preadipocytes but also for that of preadipocyte maturation to adipocytes.

## Results

### Impaired adipocyte differentiation in Psmb8^−/−^ mice

**Figure 1:**
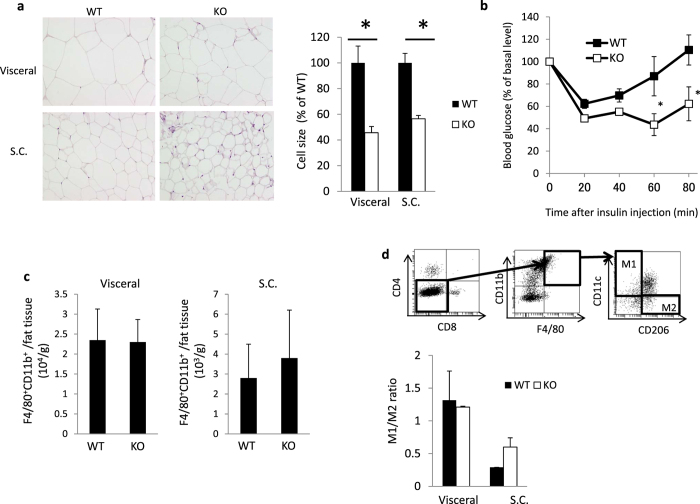
Impaired adipocyte differentiation in Psmb8^−/−^ mice. (**a**) The visceral and subcutaneous adipose tissue from WT or Psmb8^−/−^ mice at the age of 25 weeks was evaluated by HE staining. The area of 150 adipocytes was calculated and presented as means ± SD. *p < 0.05. (**b**) WT and Psmb8^−/−^ mice at the age of 25 weeks were treated with insulin (0.75 U/kg of mouse body weight). The change of blood glucose was measured every 20 min for an 80 min period after insulin injection. The data are shown as means ± SD. *p < 0.05. (**c**) The macrophages in visceral or subcutaneous adipose tissues were purified, and the number of CD11b^+^ F4/80^+^ cells was evaluated by flow cytometry. The data are shown as means ± SD. (**d**) CD11c^+^ CD11b^+^ F4/80^+^ or CD11b^+^ F4/80^+^ CD206^+^ cells were determined as M1 or M2 macrophages, respectively. The ratio of M1/M2 was calculated, and presented as means ± SD. The data in these figures are representative of three independent experiments.

The characteristic feature of JASL patients is progressive lipodystrophy^6^. We first evaluated the sizes of adipocytes in wild-type (WT) and Psmb8^−/−^ mice (Fig. 1a). Visceral and subcutaneous adipocytes were smaller in Psmb8^−/−^ mice than in WT mice (Fig. 1a). As adipocyte volume is associated with insulin sensitivity, we tested the insulin response in WT and Psmb8^−/−^ mice. Psmb8^−/−^ mice exhibited a relatively low glucose level compared with the WT mice 60 and 80 min after insulin injection, indicating that Psmb8^−/−^ mice have increased sensitivity to insulin (Fig. 1b). Although insulin signaling activates the AKT pathway^11^, AKT phosphorylation after insulin injection was comparable in adipose tissues from WT and Psmb8^−/−^ mice (Supplementary Fig. 1a), suggesting that the pathways downstream from AKT are altered in Psmb8-deficient cells. Signs of inflammation, including cell infiltration or fibrosis, were not observed in adipose tissue from either the WT or Psmb8^−/−^ mice (data not shown). Nevertheless, as there are close communications between macrophages and adipose tissue^12^,^13^, we evaluated the infiltration of macrophages (CD11b^+^ F4/80^+^ cells) (Fig. 1c) and the ratio of M1 (CD11b^+^ F4/80^+^ CD11c^+^) to M2 macrophages (CD11b^+^ F4/80^+^ CD206^+^) (Fig. 1d). However, we found no differences in these measurements between Psmb8^−/−^ and WT mice (Fig. 1d). These results support the notion that Psmb8 directly regulates the differentiation of adipocytes, rather than affecting other types of cells relevant to adipocyte differentiation or maintenance.

### Slow weight gain in Psmb8^−/−^ mice

**Figure 2:**
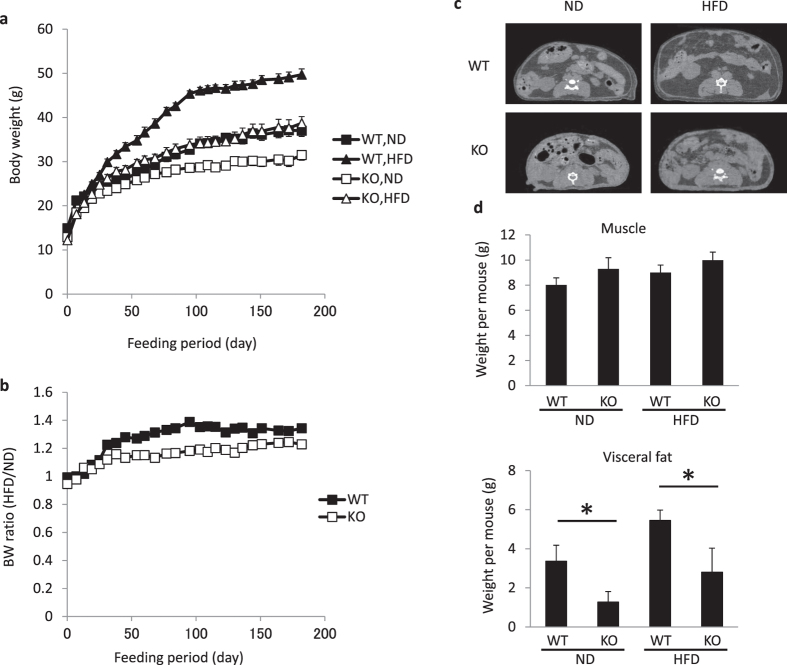
Psmb8^−/−^ showed less weight gain. WT and Psmb8^−/−^ mice were fed a normal diet (ND) or a high fat diet (HFD) from 8 to 34 weeks of age. Body weights (**a**) on the indicated day and the ratio of body weight (**b**) in mice fed with ND or HFD are exhibited. (**c**) WT or Psmb8^−/−^ mice fed with ND or HFD were evaluated by computed tomography. The computed tomographic images (at the bottom of left kidney) are shown. The areas of muscle or adipose tissue were evaluated (**d**) and the data are shown as means ± SD. *p < 0.05. The data in these figures are representative of three independent experiments.

To determine whether the smaller adipocytes in Psmb8^−/−^ mice have affected weight gain, we measured the weight of WT and Psmb8^−/−^ mice fed normal or high-fat diets from 8 to 34 weeks of age (Fig. 2a). The weight gain was much slower in the Psmb8^−/−^ mice on both diets (Fig. 2a). The ratio of body weights when the mice were fed a normal diet or high fat diet was lower in Psmb8^−/−^ than in WT mice (Fig. 2b). To evaluate whether the lower weight gain was associated with adipocyte volume, we measured the volume of muscle and adipose tissues by CT (Fig. 2c,d). The volume of muscle in Psmb8^−/−^ mice was similar to that in WT mice, whereas the volume of adipose tissue was much smaller in Psmb8^−/−^ mice than in the WT (Fig. 2c,d). The serum non-esterified fatty acids and leptin levels were lower in Psmb8^−/−^ mice than in WT that were fed with a normal diet or a high fat diet (Supplementary Fig. 1b). However, the triglyceride levels were comparable between those two mouse strains (Supplementary Fig. 1b). These data suggest that the deficiency of Psmb8 reduced adipose tissue volume, which resulted in a smaller weight gain.

### Impaired differentiation of preadipocytes during cultivation of 3T3-L1 cells

**Figure 3:**
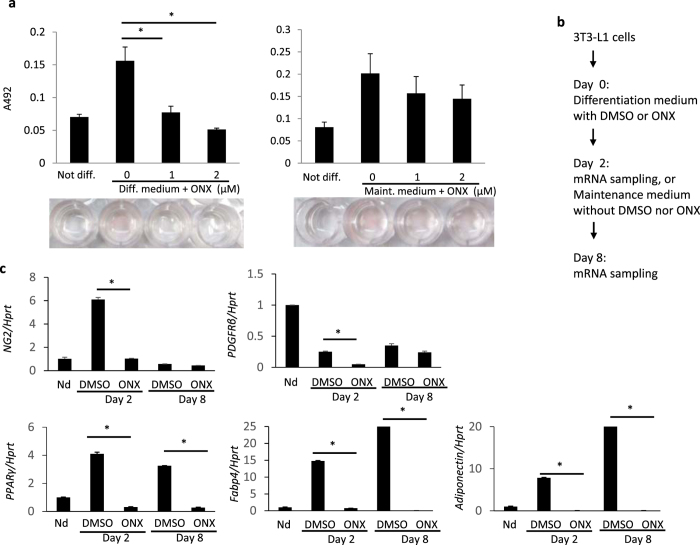
Psmb8 was required for the induction phase in 3T3-L1 cells. 3T3-L1 cells were cultured in differentiation medium containing dexamethasone (2.5 μM), 3-isobutyl-1-methylxanthine (0.5 mM) and insulin (10 μg/mL) for two days, and then cultured in maintenance medium with insulin (10 μg/mL) for the following six days. (**a**) ONX 0914 was added to the differentiation medium (left) or the maintenance medium (right) at the indicated concentrations. Cells that were fully differentiated into adipocytes were stained with Oil Red O. Oil Red O was evaluated by measuring the absorbance of the eluate. *p < 0.05. (**b**) Protocol for (**c**). (**c**) ONX 0914 was added during the first two days, cells were washed and the resultant cells cultured for another six days. The expression levels of *Ng2*, *Pdgfrb*, *Pparg*, adiponectin and *Fabp4* were evaluated by real-time PCR. The data are shown as means ± SD. *p < 0.05. The data in these figures are representative of three independent experiments.

In order to evaluate which stages of adipocyte differentiation required Psmb8 activity, we inhibited immunoproteasome activity using ONX0914, a Psmb8 inhibitor, in different stages of a 3T3-L1 culture system. The 3T3-L1 preadipocytes differentiated toward mature adipocytes when cultured with insulin and dexamethasone (differentiation medium) during the first two days, and with insulin alone (maintenance medium) during the following six days. Addition of ONX0914 during the first two days inhibited differentiation toward adipocytes as evaluated by the absorbance value of each well (Fig. 3a) and quantification of Oil Red contents (Supplementary Fig. 2a). Similar suppressive effects were observed following treatment with MG132, which is a pan-proteasome inhibitor (Supplementary Fig. 2a,b). Blockade of Psmb8 after the first two days of culture tended to suppress differentiation, although this was not statistically significant (Fig. 3a). The concentrations of ONX0914 and MG132 used in these studies were not toxic for 3T3-L1 cells (Supplementary Fig. 2c) and they allowed 3T3-L1 cells to accumulate ubiquitin-coupled proteins (Supplementary Fig. 2d). The treatment of 3T3-L1 with ONX0914 or MG132 did not induce the expression of TNF-α (Supplementary Fig. 2e).

We evaluated markers of preadipocytes and mature adipocytes in 3T3-L1 cells cultured in the presence or absence of ONX0914 during the first two days (Fig. 3b). *Ng2* and *Pdgfrb* are expressed throughout maturation from the preadipocyte stage to mature adipocytes. Expression of *Ng2* increased during the first two days of culture, and then it decreased during the following six days in the absence of ONX0914 (Fig. 3c). The initial upregulation of *Ng2* was suppressed by addition of ONX0914, and the *Ng2* level remained low during the following six days (Fig. 3c). Expression of *Pdgfrb* was downregulated during the first two days of culture in the absence of ONX0914, and its level was further reduced in the presence of ONX0914 (Fig. 3c). The reduced expression of NG2 and PDGFRβ proteins was also observed after a 2-day treatment with ONX0914 (Supplementary Fig. 3a,b). The expression of an adipogenic gene, *Cebpa*, and the gene product, C/EBPα was also lower by the treatment of 3T3-L1 cells with ONX0914 or MG132 (Supplementary Fig. 3c). *Pparg*, *Fabp4* and *adiponectin* are markers for mature adipocytes. These three genes were upregulated during the first two days of culture in the absence of ONX0914. In contrast, ONX0914 addition during the first two days blocked the upregulation of all three molecules, and their levels remained low during the next six days (Fig. 3c). These data demonstrate that Psmb8 activity directly regulates the differentiation of preadipocytes to mature adipocytes.

### Reduced numbers of preadipocytes and impaired differentiation of preadipocytes in Psmb8^−/−^ mice

**Figure 4:**
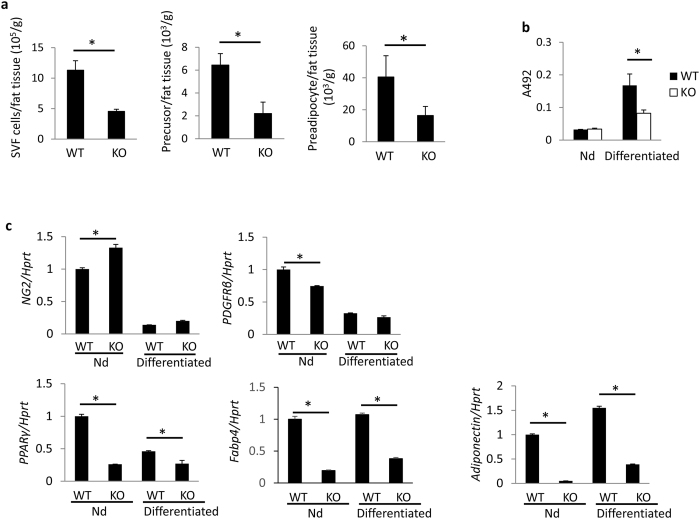
Psmb8 was required for preadipocyte differentiation. The cells from SVF were analyzed by staining with anti-CD24, anti-CD31, anti-CD34, anti-CD29, anti-CD45 and anti-Sca1 antibodies and analyzed by flow cytometry. (**a**) The cell numbers of SVF, CD45^−^CD31^−^CD29^+^ CD34^+^ Sca1^+^ CD24^−^ or CD45^−^CD31^−^CD29^+^ CD34^+^ Sca1^+^ CD24^+^ from WT and Psmb8^−/−^ (KO) mice were counted. The data are shown as means ± SD. *p < 0.05. (**b**) CD24^+^ CD34^+^ cells from WT (WT) or Psmb8^−/−^ (KO) mice were cultured in the presence of dexamethasone, 3-isobutyl-1-methylxanthine and insulin for two days, and cultured with insulin for another six days. Cells were stained with Oil Red O, and the OD492 value was measured. The data are shown as mean ± SD. *p < 0.05. (**c**) The expression levels of *Ng2*, *Pdgfrb*, *Fabp4*, *Pparg* and adiponectin were evaluated using real-time PCR after two days’ culture with dexamethasone, 3-isobutyl-1-methylxanthine and insulin, and a further six days’ culture with insulin. The data are shown as means ± SD. *p < 0.05. The data in these figures are representative of three independent experiments.

The preadipocyte and its precursor are defined respectively by two immunophenotypes: CD45^−^CD31^−^CD29^+^ CD34^+^ Sca1^+^ CD24^−^ and CD45^−^CD31^−^CD29^+^ CD34^+^ Sca1^+^ CD24^+ ^14, (Supplementary Fig. 4a). We tested the number of preadipocytes and precursors in Psmb8^−/−^ mice to assess whether Psmb8 also regulated the differentiation of preadipocytes. The total number of cells in the stromal vascular fraction (SVF) was lower in Pmsb8^−/−^ mice than in control mice (Fig. 4a). The number of preadipocytes and its precursors were also lower in the Psmb8^−/−^ mice (Fig. 4a), indicating that the lack of Psmb8 affected preadipocyte differentiation itself. The expression of *Cebpa* was lower in the SVF from Psmb8^−/−^ than WT mice (Supplementary Fig. 4b). We then cultured purified preadipocytes in the presence of dexamethasone and insulin for two days, and then insulin alone for the next six days. The maturation of adipocytes evaluated using Oil Red O staining was suppressed in the Psmb8^−/−^ mice compared with the control mice (Fig. 4b and Supplementary Fig. 4c). The mRNA levels of the preadipocyte markers *Ng2* and *Pdgfrb* were similar in WT and Psmb8^−/−^ mice before *in vitro* culture, and the expression levels of both genes were reduced after differentiation. However, both before and after cell culture the expression levels of *Pparg*, *Fabp4* and *Adiponectin*, which are markers of mature adipocytes, were lower in Psmb8^−/−^ cells than in control cells (Fig. 4c).

## Discussion

The missense mutation in *PSMB8* disturbs the functions of immunoproteasomes, which in turn causes lipodystrophy. We have found that the mutation is directly involved in the differentiation of adipocytes^6^. However, it remains unclear how and when immunoproteasomes affect adipocyte differentiation. Psmb8^−/−^ mice were reported more than 20 years ago^15^, but a defect of adipocyte differentiation in these mice has not been reported. We have demonstrated here that Psmb8^−/−^ mice show slower weight gain than control mice and have a reduced adipocyte volume. Psmb8^−/−^ mice have a reduced number of preadipocyte precursors and preadipocytes. Furthermore, preadipocytes from Psmb8^−/−^ mice have a reduced ability to fully mature. These data indicate that Psmb8-mediated immunoproteasome activity is a crucial regulator not only for the differentiation of preadipocytes to mature adipocytes, but also for the differentiation of preadipocyte precursors.

Adipocyte differentiation was mimicked in 3T3-L1 cells in this study *in vitro*. The differentiation was induced by dexamethasone and insulin for two days, and the process was then maintained by insulin for the next six days, a procedure that generates terminally mature adipocytes. Blockade of Psmb8 during the first two days suppressed the differentiation toward mature adipocytes. When the blockade was limited to the final six days, the treatment also tended to inhibit the generation of mature adipocytes. Therefore, Psmb8 appears to be required for the differentiation process from preadipocytes to mature adipocytes.

What is the mechanism of Psmb8-mediated adipocyte differentiation? One possibility is that blockade of the degradation of ubiquitin-coupled protein ‘damages’ cells, thereby disturbing the differentiation of the cells. Although we have reported that downregulation of Psmb8 in 3T3-L1 cells does not affect cell viability itself^6^, other types of ‘damage’ such as ER stress or excessive accumulation of ubiquitin-coupled protein might be responsible for the disturbance. We and another group^16^ demonstrated that the treatment of 3T3-L1 with a PMSB8-specific inhibitor or a pan-chymotrypsin inhibitor blocked differentiation toward adipocytes. Thus, another possibility is that blockade of chymotrypsin-like activity specifically affects the expression of proteins that are essential for adipocyte differentiation.

Several studies have demonstrated that a subpopulation of adipocyte progenitors (Lin^−^:CD29^+^:CD34^+^:Sca-1^+^:CD24^+^) in the SVC fraction from adipose tissue differentiates into an adipose depot *in vivo*^17^. Subsequent studies have revealed that CD24^+^ cells are precursors for generating the CD24^−^ preadipocyte population^14^. The total number of preadipocyte precursors and preadipocytes was smaller in Psmb8^−/−^ mice than in WT mice, which demonstrates that differentiation toward preadipocyte precursors is impaired in the absence of Psmb8. Consistent with data obtained from the 3T3-L1 cell culture system, differentiation from preadipocytes to mature adipocytes was suppressed in Psmb8^−/−^ cells. It remains unclear whether Psmb8 is required *in vivo* for preadipocytes or for maintaining the environment of preadipocyte differentiation or for both functions. Nonetheless, impairment of preadipocytes or adipocyte differentiation would contribute to the lower adipose tissue volume in Psmb8^−/−^ mice.

Lipodystrophy, which is characterized by partial or complete loss of adipose tissue, occurs in many diseases including dyslipidemia and insulin-resistant diabetes. On the other hand, a single gene mutation can also cause lipodystrophy^18^. For instance, complete lipodystrophy is caused by mutations in caveolin-1 (*CAV*), seipin (*BSCL2*) or *AGPAT2*^18^. Mutations in PPARγ (*PPARG*) or lamin A/C (*LMNA*) can cause partial lipodystrophy. These causative genes are involved in various stages of adipogenesis. The *PSMB8* mutation in JASL causes partial lipodystrophy especially in the upper or lower extremities. Thus, the contribution of *PSMB8* to adipocyte differentiation may vary depending on the site. In any case, distinguishing between *PSMB8* mutations and other altered genes that contribute to lipodystrophy might shed light on the pathophysiology of lipodystrophy.

Patients with a *PSMB8* mutation are subject to autoinflammatory symptoms, including high fever and nodular erythema^6^ whereas mice that lack *Psmb8* do not exhibit similar inflammatory responses. The mechanisms underlying the different phenotypes remain unclear. One possibility is that strong compensatory mechanisms are induced in mice. For example, we found upregulated *Psmb5* expression in cells from *Psmb8-*deficient mice (data not shown). As for the association of lipodystrophy and inflammation, lipodystrophy is progressive even when inflammation is slowed by immunosuppressive drugs. Those data also support the notion that lipodystrophy in patients with a *PSMB8* mutation is mainly attributable to the impaired adipocyte differentiation.

## Methods

### Mice

C57BL/6 mice were purchased from Japan SLC (Hamamatsu, Japan). Psmb8^−/−^ mice^15^ were maintained under specific pathogen-free conditions in the animal research center of Tokushima University, and all animal experiments were approved by the animal research committee of Tokushima University and performed in accordance with our institution’s guidelines for animal care and use.

### Flow cytometry

Cells were detached by incubation in 10 mM EDTA-PBS for 10 min and resuspended in staining buffer at a density of 2 × 10^6^ cells/mL. The cells were incubated with anti-*Fcγ* RII/III monoclonal antibody (mAb) (2.4G2), followed by antibodies against CD29 (HMβ1-1), CD31 (390), CD34 (MEC14.7), CD45 (A20), Sca-1(D7), CD24 (M1/69), F4/80 (BM8), CD11b (M1/70), CD11c (N418), CD206 (C068C2), CD4 (GK1.5), CD8 (53–6.7) and PDGFRβ (APB5) obtained from BioLegend (San Diego, CA). Anti-NG2 antibody (AB5320) was obtained from Merck Millipore (Darmstadt, Germany). After gating out cells that were positive for 7-AAD, the fluorescence intensity of 10^5^ cells was measured with a FACSCanto II flow cytometer (BD Biosciences, CA) and analyzed with FACSDiva (BD Biosciences) or FlowJo (Tree Star) software programs. CD11c^+^ CD11b^+^ F4/80^+^ or CD11b^+^ F4/80^+^ CD206^+^ cells were determined as M1 or M2 macrophages, respectively, and the numbers of each type of macrophage were calculated by the percentages and total cell number to indicate the M1/M2 ratio. CD45^−^ CD31^−^ CD29^+^ CD34^+^ Sca1^+^ CD24^−^ or CD45^−^ CD31^−^ CD29^+^ CD34^+^ Sca1^+^ CD24^+^ cells were determined as preadipocytes or adipocyte precursors, respectively. NG2^+^ or PDGFRβ^+^ 3T3-L1 cells were assessed after treating the cells with the inhibitors in differentiation medium for 2 days. The data are expressed as the percentage of positive cells and mean fluorescence intensity (MFI) of the peak.

### Isolation of fat tissue

For histological analysis, epididymal and subcutaneous fat tissues of 25-week-old male mice were collected and fixed in 3.7% formaldehyde to make histological sections for hematoxylin and eosin staining. The size of each adipocyte was measured with ImageJ, which provided the average size of 150 cells of each group.

### Insulin tolerance test

For the insulin tolerance test, blood glucose concentrations of mice (n = 3) were determined with Glucose Pilot blood glucose test strip (Obelis S. A., Brussels, Belgium) before and 20, 40, 60 and 80 min after intraperitoneal injection of 0.75 U/kg insulin (Eli Lilly Japan K.K.).

### High fat diet experiments

For the high-fat diet experiment, eight-week-old mice (n = 5) were fed with a control diet (MF diet, Oriental Yeast Co., Tokyo, Japan) or a high fat diet (HFD-60; 60 kcal% fat, 33 kcal% lard and 5.5 kcal% sucrose, Oriental Yeast Co., Ltd.). Body weights were recorded two or three times each week during the experiment. The mice were anesthetized with an intraperitoneal injection of chloral hydrate and then analyzed with a CT scanning machine (Latheta LCT-200, Hitachi Aloka Medical, Tokyo, Japan) to determine the weights of muscle and fat tissues. To estimate the weights of these organs, volume (cm^3^) was multiplied by density (cm^3^/g) which is 1.06 or 0.92 for muscle or fat tissue, respectively, according to a manual for the CT machine.

### Blood analysis

Plasma triglyceride and non-esterified fatty acids levels were determined enzymatically using LabAssay Triglyceride and LabAssay NEFA, respectively, from Wako Pure Chemical Industries (Osaka, Japan). Serum leptin was measured using Mouse/Rat Leptin ELISA (BioVendor, Karásek, Czech Republic).

### Isolation of stromal vascular fraction (SVF) cells

Epididymal and subcutaneous fat tissues were harvested, weighed, and cut into small pieces with scissors, following incubation in adipose isolation buffer^19^ containing 1 mg/mL collagenase (Wako Pure Chemical Industries) for one hour at 37 °C with gentle shaking. SVF cells were collected as a pellet by centrifugation at 500 × g for 5 min at 4 °C and washed with PBS before counting the number of cells.

### Cell culture

3T3-L1 cells or SVF cells were cultured in DMEM (Wako Pure Chemicals Industries) supplemented with 10% FBS and penicillin/streptomycin solution (Gibco) and maintained in a humidified incubator at 37 °C and 5% CO_2_. During the cultivation of SVF cells, unattached cells were removed by pipetting when changing the medium. Two days after reaching confluence, cells were incubated in the differentiation medium (AdipoInducer Reagent (for animal cells); Takara Bio Inc., Shiga, Japan) containing dexamethasone (2.5 μM), 3-isobutyl-1-methylxanthine (0.5 mM) and insulin (10 μg/mL) for two days. The medium was then replaced with the maintenance medium that included 10 μg/mL insulin in 10% FBS-DMEM with antibiotics. The maintenance medium was renewed every two or three days during six days of culture. The PSMB8-specific inhibitor ONX0914 (Adooq Bioscience, CA) or a proteasome inhibitor MG132 (LifeSensors, PA) was dissolved in DMSO and added to the differentiation or maintenance medium at the indicated concentration. Adipocyte differentiation was determined by Oil Red O staining. The stained cells were observed by microscopy (Diaphot 300, Nikon, Tokyo, Japan), followed by evaluating stained area with ImageJ. Oil Red O uptake into the cells was quantified by extraction with isopropanol, and measurement of the absorbance of the eluate at 492 nm. Cell toxicity of the inhibitors was evaluated by counting the number of 3T3-L1 cells using trypan blue after incubating the cells with the inhibitors for 1 day.

### Real-time PCR

Total RNA of differentiated or undifferentiated adipogenic cells was isolated with TRIzol reagent (Thermo Fisher Scientific Inc., MA), and cDNA was synthesized (300 ng of RNA) with ReverTra qPCR RT Master Mix with gDNA Remover (Toyobo Co., Osaka, Japan). Relative expression was calculated using the comparative threshold cycle method. qRT-PCR analyses were performed by using StepOnePlus (Applied Biosystems) with the following primers : *Ng2*, F: AGGCGTCTACCGATGTGATGT, *Ng2,* R: TGGCTGCCCTGTAGTGAAAC; *Pdgfrb,* F: AGACACTGGGGAATACTTTTGTG, *Pdgfrb*, R: CGGCCCTAGTGAGTTGTTGT; *Pparg,* F: GTGCCAGTTTCGATCCGTAGA, *Pparg,* R: GGCCAGCATCGTGTAGATGA; *Adiponectin*, F: GCACTGGCAAGTTCTACTGCAA, *Adiponectin*, R: GTAGGTGAAGAGAACGGCCTTGT; *Fabp4,* F: GAATTCGATGAAATCACCGCA, *Fabp4,* R:CTCTTTATTGTGGTCGACTTTCCA; *Cebpa*, F: CAAGAACAGCAACGAGTACCG, *Cebpa*, R: GTCACTGGTCAACTCCAGCAC; *Tnfa*, F: CTGTAGCCCACGTCGTAG, *Tnfa*, R: TTGAGATCCATGCCGTTG; *Hprt,* F: AGCCTAAGATGAGCGCAAGT, *Hprt,* R: TTACTAGGCAGATGGCCACA. The mRNA level of *Hprt* was used as an internal control.

### SDS-PAGE and Western blotting

Whole cell lysates were obtained in lysis buffer consisting of 150 mM NaCl, 10 mM Tris-HCl (pH7.4), 1 mM EGTA, 1 mM EDTA, 1% Triton X-100 and protease inhibitors (Roche, Mannheim, Germany). Protein concentration was determined using a Pierce BCA protein assay kit (Thermo Fisher Scientific Inc., MA), and 50 μg of protein for each sample was applied to a 10% acrylamide gel for SDS-PAGE. The proteins were transferred to a PVDF membrane, followed by blocking with a 5% skim milk solution, and then reacted with primary antibodies. The antibodies used for Western blotting were as follows: ubiquitin (ab19247, 1:2,000; Abcam, Cambridge, UK); Akt (4691P) and p-Akt (4060P) (Cell Signaling Technology, Danvers, MA); C/EBPα (sc-61) (Santa Cruz Biotechnology, Santa Cruz, CA); β-actin (A2103; Sigma-Aldrich, St. Louis, MO), anti-rabbit IgG labeled with horseradish peroxidase (170–6515; Bio-Rad, Hercules, CA). An ECL Kit (GE Healthcare, Little Chalfont, UK) was used to visualize the protein bands, and ImageJ for quantification of the band intensity.

### Statistical analysis

The statistical significance of between-group differences was evaluated by an unpaired, two-tailed t test. A p value of <0.05 was considered significant.

## Additional Information

**How to cite this article**: Arimochi, H. *et al.* Differentiation of preadipocytes and mature adipocytes requires PSMB8. *Sci. Rep.* **6**, 26791; doi: 10.1038/srep26791 (2016).

## Supplementary Material

Supplementary Information
