# Supplementary material for: Differentiation of preadipocytes and mature adipocytes requires PSMB8
Source: Sci Rep. 2016 May 26;6:26791. doi: 10.1038/srep26791 (PMC4880908; doi:10.1038/srep26791)
Supplement: Supplementary Information [file srep26791-s1.pdf]

Differentiation of preadipocytes and mature adipocytes requires PSMB8

Hideki Arimochi, Yuki Sasaki, Akiko Kitamura, Koji Yasutomo

Supplemental Figure 1. Arimochi *et al.*

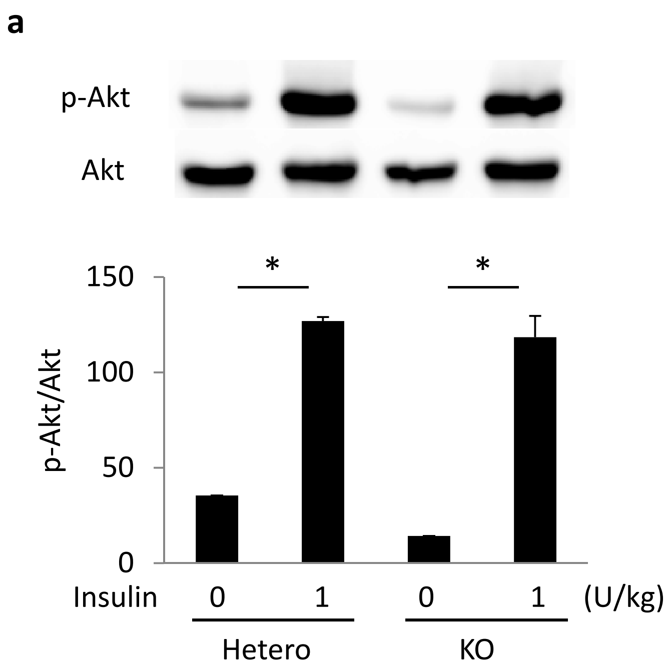

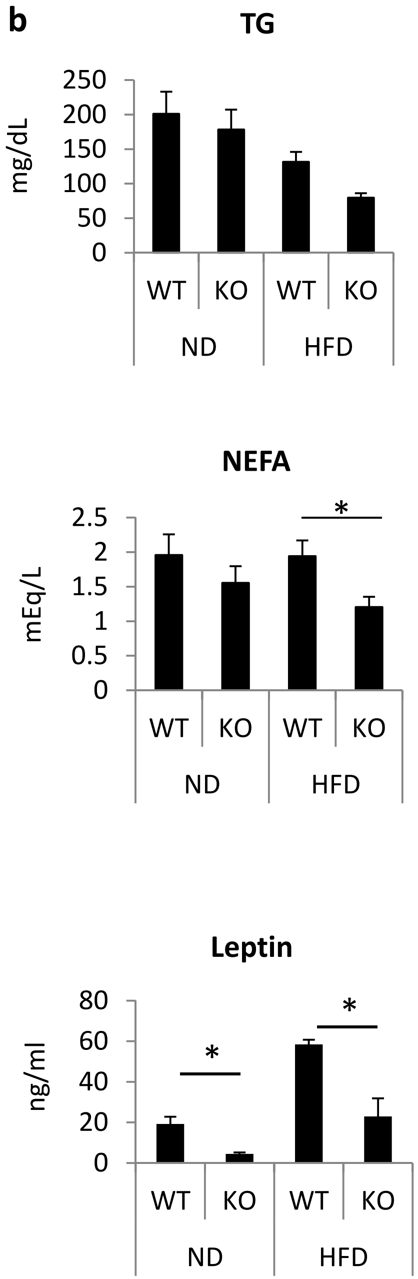

Supplemental Figure 2. Arimochi *et al.*

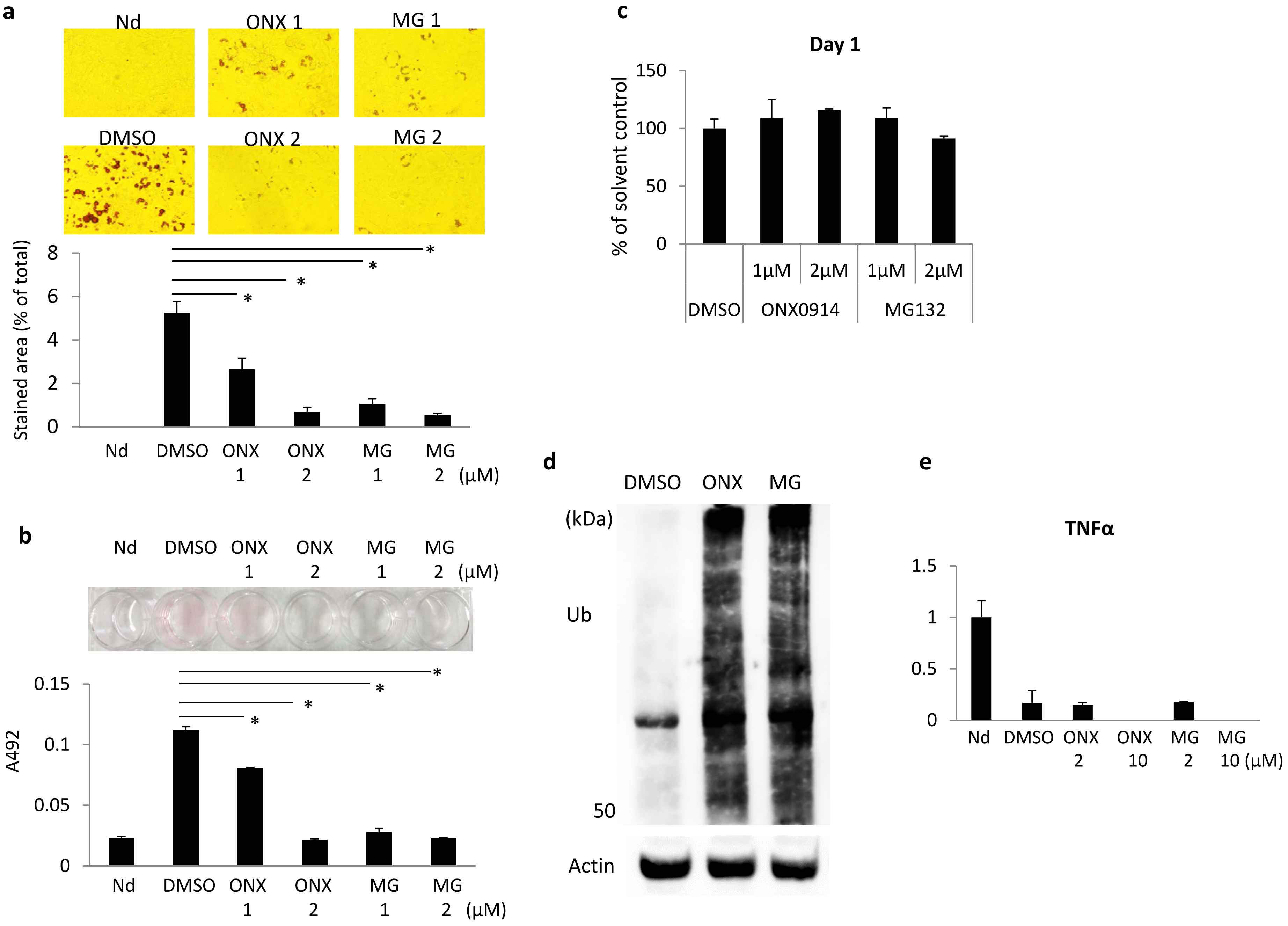

Supplemental Figure 3. Arimochi *et al.*

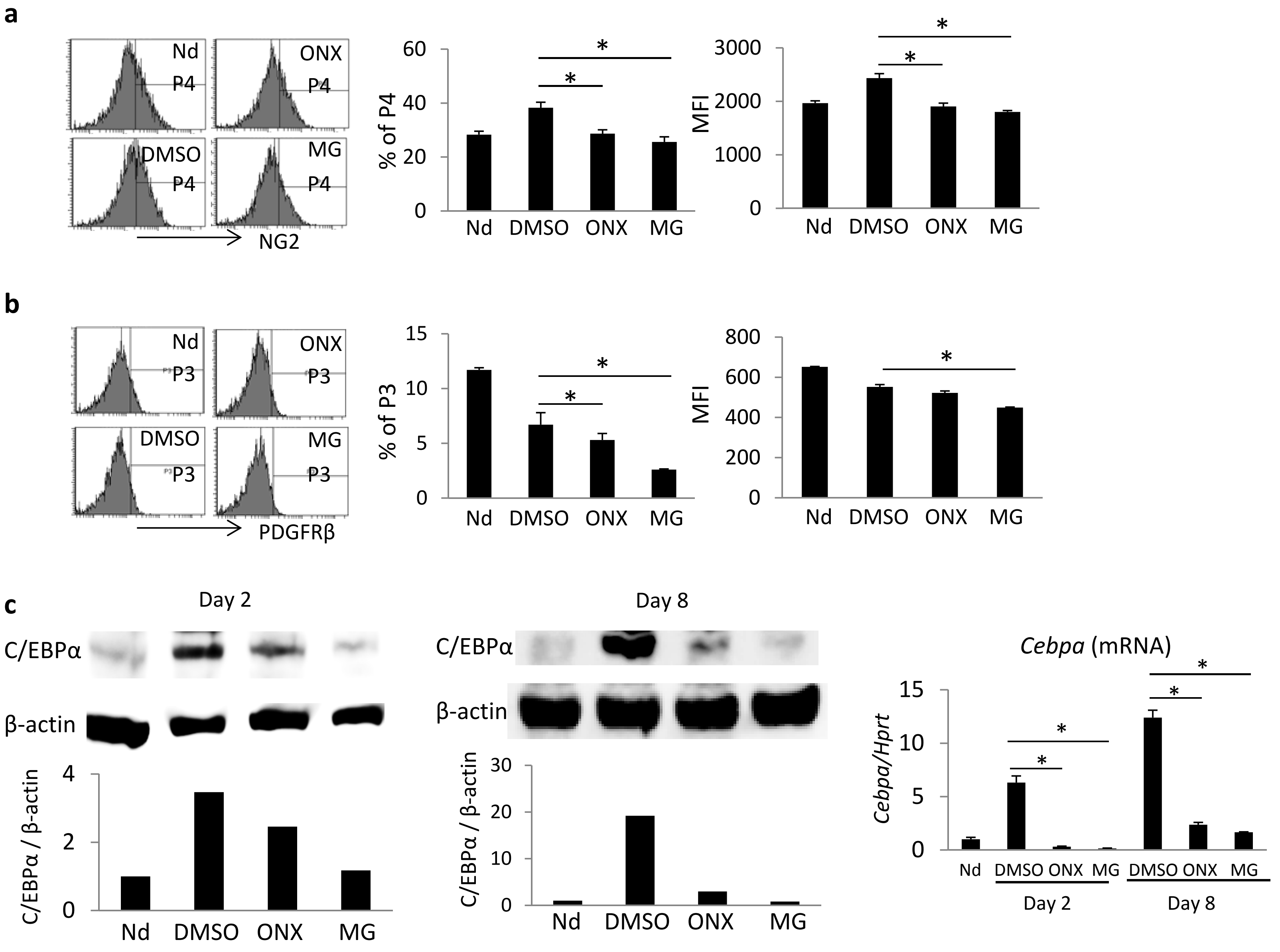

Supplemental Figure 4. Arimochi *et al.*

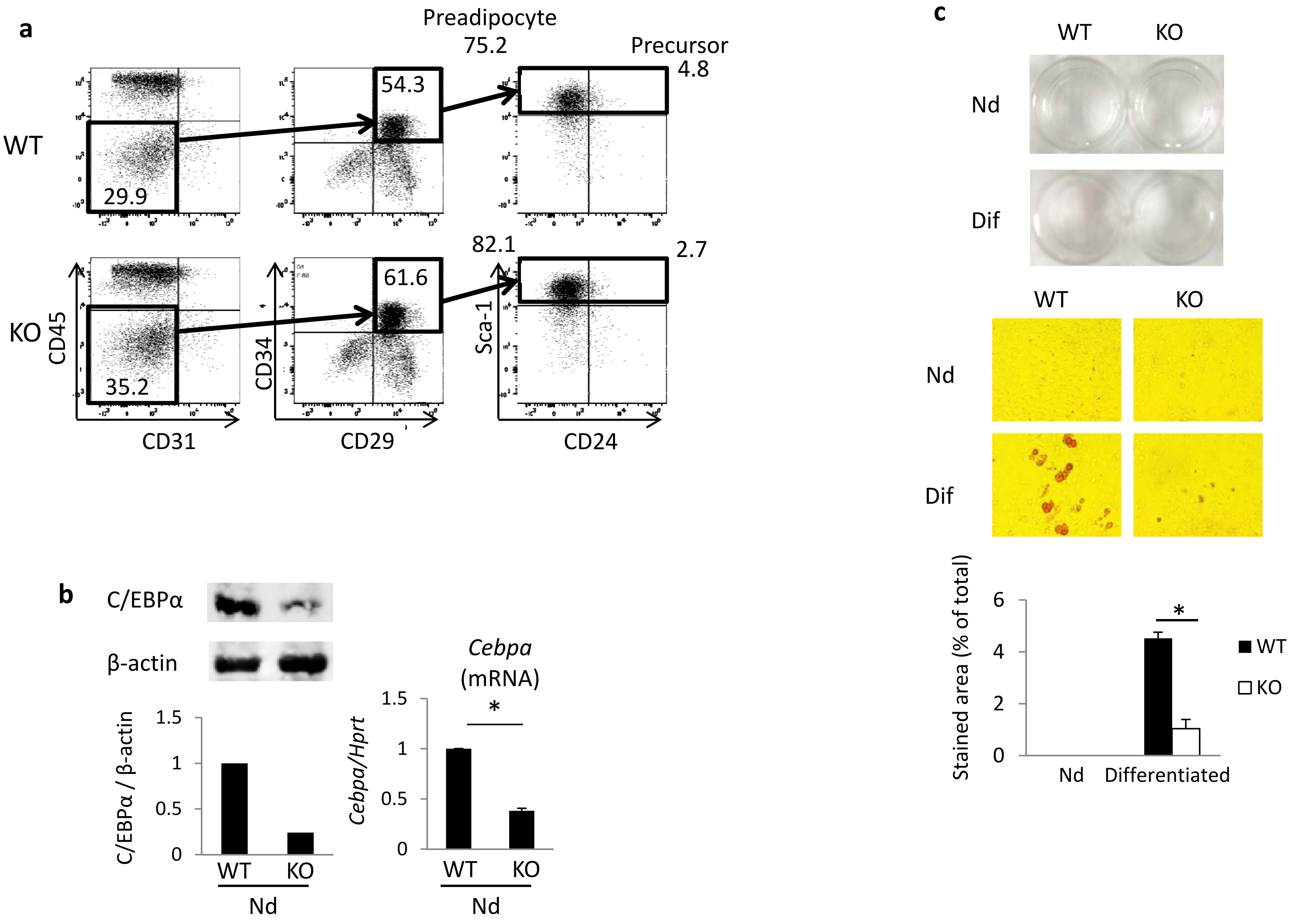

Supplemental Figure Legends

Supplemental Figure 1. Psmb8-/- showed higher insulin sensitivity and lower serum leptin levels

(a) WT and Psmb8-/- mice were fasted overnight and were then injected intraperitoneally with insulin (1 U/kg of mouse body weight). Visceral adipose tissue of the mice 10 min after the injection was used to determine phosphorylated Akt levels by Western blotting and image analysis. The data are shown as means ± SD. *, $p < 0.05$. (b) Mice were fed a normal diet (ND) or a high fat diet (HFD) from 8 to 34 weeks of age. Plasma triglyceride (TG), non-esterified fatty acids (NEFA) and leptin levels were determined. The data are shown as means ± SD. *, $p < 0.05$. The data in these figures are representative of three independent experiments.

Supplemental Figure 2. Proteasome inhibitors suppressed adipocyte differentiation in 3T3-L1 cells

3T3-L1 cells were cultured in differentiation medium containing ONX0914 or MG132 for two days, and then cultured in maintenance medium without the inhibitors for the following six days. Cells were differentiated into adipocytes and then stained with Oil Red O. (a) The stained cells were observed by microscopy and the stained area was

evaluated with Image J image analysis software. The data are shown as means ± SD. *, $p < 0.05$. (b) Oil Red O was evaluated by measuring the absorbance of the eluate. The data are shown as mean ± SD. *, $p < 0.05$. (c) 3T3-L1 cells were cultured with the inhibitor for 1 day. The number of cells was counted using trypan blue, and cell viability was calculated. The data are shown as means ± SD. (d) 3T3-L1 cells were cultured with ONX0914 (2 μM) or MG132 (2 μM) for 12 h. Ubiquitinated protein was detected by Western blotting. (e) 3T3-L1 cells were cultured in differentiation medium for two days, and then cultured in maintenance medium with the inhibitor for another six days. *Tnfa* expression level was evaluated by real-time PCR. The data are shown as means ± SD. The data in these figures are representative of three independent experiments.

Supplemental Figure 3. Proteasome inhibitors changed protein expression levels related to adipocyte differentiation in 3T3-L1 cells

The 3T3-L1 cells were cultured in differentiation medium containing 2 μM ONX0914 or 2 μM MG132 for two days (Day 2), and then cultured in maintenance medium without the inhibitors for the following six days (Day 8). Protein expression levels of NG2 (a) and PDGFRβ (b) were determined on Day2 by flow cytometry. The data are shown as means ± SD. *, $p < 0.05$. (c) Protein and mRNA expression levels of C/EBPα

were determined on Days 2 and 8 by Western blotting and real-time PCR, respectively. Nd: not differentiated. The data are shown as mean ± SD. *, $p < 0.05$. The data in these figures are representative of three independent experiments.

Supplemental Figure 4. Psmb8 was required for preadipocyte differentiation of SVF cells

(a) The cells in SVF were analyzed by staining with anti-CD24, anti-CD31, anti-CD34, anti-CD29, anti-CD45 and anti-Sca1 antibodies and analyzed by flow cytometry. (b) Protein and mRNA expression levels of C/EBPα were determined by Western blotting and real-time PCR, respectively. Nd: not differentiated. The data are shown as means ± SD. *, $p < 0.05$. (c) SVF cells were cultured in differentiation medium for two days and maintenance medium for the following six days, and then stained with Oil Red O. The stained cells were observed by microscopy and the stained area was evaluated with Image J image analysis software. The data are shown as means ± SD. *, $p < 0.05$. The data in these figures are representative of three independent experiments.
